# Building an octaploid genome and transcriptome of the medicinal plant *Pogostemon cablin* from Lamiales

**DOI:** 10.1038/sdata.2018.274

**Published:** 2018-12-11

**Authors:** Yang He, Fu Peng, Cao Deng, Liang Xiong, Zi-yan Huang, Ruo-qi Zhang, Meng-jia Liu, Cheng Peng

**Affiliations:** 1 State Key Laboratory Breeding Base of Systematic Research, Development and Utilization of Chinese Medicine Resources, Chengdu University of Traditional Chinese Medicine, Chengdu, 610075, China; 2 West China School of Pharmacy, Sichuan University, Chengdu, 610041, China; 3 Departments of Bioinformatics, DNA Stories Bioinformatics Center, Chengdu, 610000, China

**Keywords:** Plant sciences, Evolution

## Abstract

The Lamiales order presents highly varied genome sizes and highly specialized life strategies. Patchouli, *Pogostemon cablin* (Blanco) Benth. from the Lamiales, has been widely cultivated in tropical and subtropical areas of Asia owing to high demand for its essential oil. Here, we generated ~681 Gb genomic sequences (~355X coverage) for the patchouli, and the assembled genome is ~1.91 Gb and with 110,850 predicted protein-coding genes. Analyses showed clear evidence of whole-genome octuplication (WGO) since the pan-eudicots γ triplication, which is a recent and exclusive polyploidization event and occurred at ~6.31 million years ago. Analyses of *TPS* gene family showed the expansion of type-a, which is responsible for the synthesis of sesquiterpenes and maybe highly specialization in patchouli. Our datasets provide valuable resources for plant genome evolution, and for identifying of genes related to secondary metabolites and their gene expression regulation.

## Background & Summary

*Lamiales* (eudicots/Asterids I clade), one of the largest orders of flowering plants, has more than 23,000 species in at least 23 families^1^ and representatives are found all over the world. Well-known, economically or medicinally important members of this order include lavender, olive^2^, the ash tree^3^, sesame^4^, bladderwort^5^, mint^6^ and patchouli^7^. *Lamiales* species present highly specialized life strategies, such as carnivory, parasitism, epiphytism, and desiccation tolerance^8^. Some lineages possess drastically miniaturized genomes (for example, 77 Mb for *Utricularia gibba*^5^), while some are relatively large (for example, 5,537 Mb for *Lavandula officinalis* Chaix^9^), although different levels of polyploidization occurred since they diverged from their common ancestor^2–5,10^. Therefore, it’s of great importance to explore the evolution of the *Lamiales* at the genome level.

Patchouli, *Pogostemon cablin* (Blanco) Benth., is one of species from the *Lamiaceae* (mint family), and has been widely cultivated in tropical and subtropical areas of Asia owing to high demand for its essential oil^11^. Chemical and pharmacological studies of patchouli over the last few decades indicated that the patchouli oil contains >40 major components^7^. These compounds play an essential role in plant reproduction, defence, and signalling, and are also precious important industrial ingredient for perfumes, incense, soaps and cosmetic products^12,13^. In addition, these constituents exhibit marked activities, such as antibacterial, anti-influenza virus, anti-inflammatory, cytotoxic, antimutagenic, anti-PAF–induced platelet aggregation, insecticidal, and hepatoprotective activities^7^; therefore, have therapeutic roles in heat and dampness elimination, nerve smoothness and fatigue alleviation, indigestion, headache, and fever^14^.

*De novo* genome assembly of key species in large-scale clade could deepen our understanding of paleo-polyploidization events and chromosome evolution^15,16^. The genome of domesticated sunflower (*Helianthus annuus* L.)^15^ enabled the reconstruction of the evolutionary history of the Asterids, further establishing the existence of a whole-genome triplication (WGT) at the base of the Asterids II clade. Comparative genomics combined with functional genomics have been demonstrated as powerful toolkits to identify candidate genes responsible for particular biological characteristics^15–17^, including the biosynthesis of secondary metabolism^18–20^.

**Figure 1:**
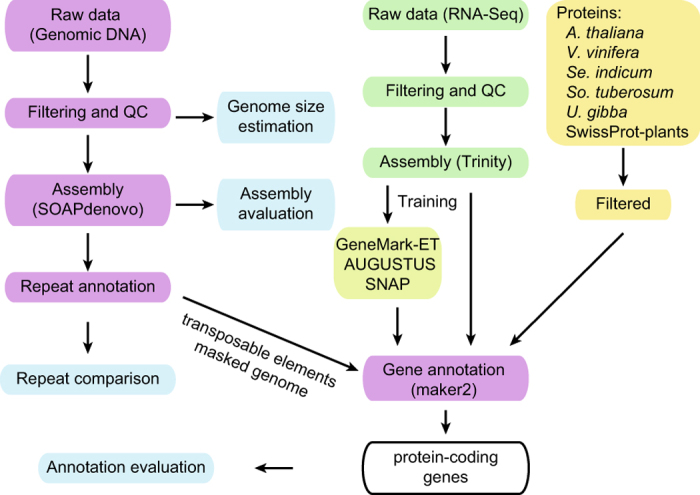
Overview of the pipeline of the study.

The genome of patchouli was sequenced and analyzed as the flowchart shown in Fig. 1. The aim of this study is to obtain genome sequences with high quality for the patchouli. The datasets reported here will be useful (1) for validating the polyploidization events in patchouli, *Lamiales*, and asterids, (2) for exploring the relationship of highly diverse palaeohistory to the highly varied genome size, highly specialized life strategies, and highly diverse species in *Lamiales,* (3) for studying the dynamic diploidization and gene retention in medicinal polyploidies, (4) for investigating the subfunctionalization after the whole-genome octuplication (WGO) in patchouli and the influence on the sesquiterpenes biosynthesis, (5) for analysing of protein-coding gene families and the regulation of their expression, such sesquiterpenes synthases and their regulators in relation to the patchouli oil biosynthesis.

## Methods

### Materials and sequencing

Single wild *P. cablin* plant individual was collected from Gaoche village, Yangchun City, Guangdong Province, China. Total 49.1 ug high quality genomic DNA was extracted from patchouli using an improved CTAB method. For *de novo* genome sequencing, whole-genome shotgun sequencing strategy was employed and short paired-end inserts (250 bp, 500 bp and 700 bp) and long mate-paired inserts (2 kb, 5 kb, and 10 kb) were constructed using the standard protocol provided by Illumina (San Diego, USA). Paired-end sequencing was performed using the Illumina HiSeq (Supplementary Table S1). Leaves, stems and roots of wild plants were collected and all samples were immediately frozen in liquid nitrogen and stored at −80 °C for later RNA sequencing (RNA-Seq). Each tissue has three biological replicates (Supplementary Table S2).

### Genome assembly

The short insert size (250 bp 500 bp and 700 bp) pair end reads were filtered by removing adaptor sequences, PCR duplicates and low-quality reads using Trimmomatic (v3.20)^21^, followed by error correction using SOAPec (v2.01)^22^. For the read-through libraries (the target DNA fragment size is less than twice the single-end read length, so that the reads may overlap. e.g., 150 bp Illumina reads taken from 250 bp insert size library), the corresponding paired-end reads were merged into a longer fragment if there exists an overlap using PEAR^23^. The long mate-pair reads (2 kb, 5 kb and 10 kb) were trimmed using NextClip^24^, and fragments with the junction adapter in at least one of the paired reads were used. The statistics of final clean reads were listed in Supplementary Table S3.

To estimate the genome size of patchouli, KmerFreq_AR program from the SOAPec (ver. 2.01 package, http://soap.genomics.org.cn/about.html), KmerGenie^25^, and Jellyfish^26^ were used to construct the k-mer frequency spectrum using multiple datasets (Supplementary Table S4 and Figure S1).

**Figure 2:**
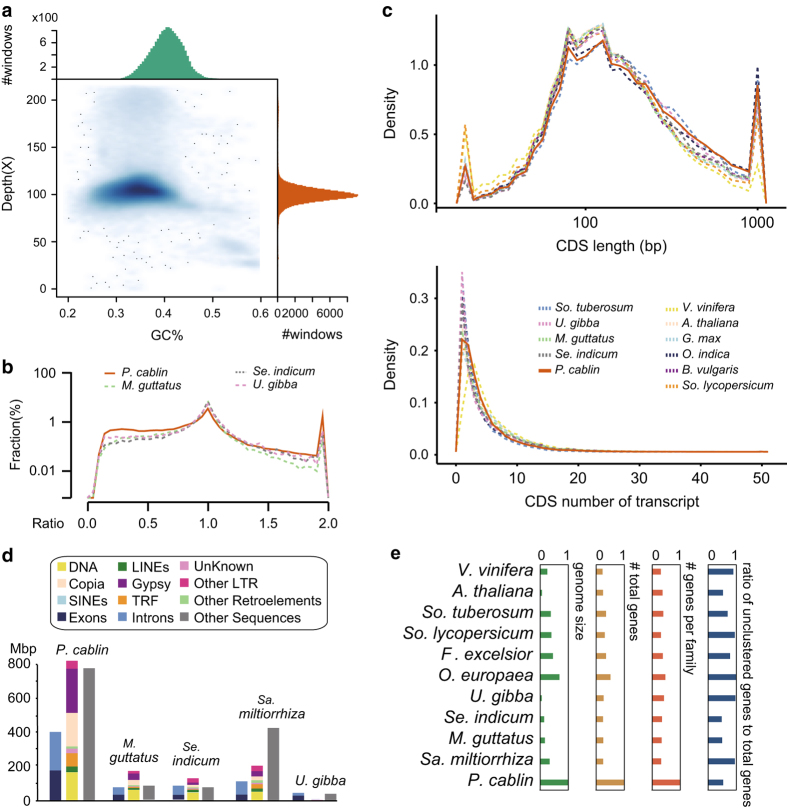
Comparison of genomic elements. (**a**) Distribution of GC contents and sequencing depths. The x-axis represents the distribution of GC contents and the y-axis represents the distribution of average depth. (**b**) Distribution of ratio of homologous pair’s lengths. The ‘homologous pair’ is the pair of two homologous proteins from two species. In our analysis, one is from the patchouli, and the other one is from the SwissProt database. ‘Ratio = (length of patchouli proteins) / (length of its SwissProt homolog). (**c**) The distribution of the CDS length and the CDS numbers of transcripts. (**d**) Comparisons of genomic elements from patchouli and other relatives. Genomic sequences were grouped into three groups, including genic (exons and introns), repetitive and other (neither genic nor repetitive region). (**e**) Comparisons of genome and gene family size among 11 species. Each category is normalized by its maximum.

Whole-genome shotgun assembly of the patchouli was performed using the short oligonucleotide analysis package SOAP*denovo2*^22^ (K = 127). Gaps were then closed using the paired-end information to retrieve read pairs in which one end mapped to a unique contig and the other was located in the gap region using GapCloser (version 1.10)^22^. The statistics of final assembly were listed in Supplementary Table S5 and the GC-depth distribution was shown in Fig. 2a. The core eukaryotic gene mapping approach (CEGMA) (Supplementary Table S6) and benchmarking universal single-copy orthologs (BUSCO) methods were used to assess assembly quality.

### Repeat annotation

*De novo* repeat annotation of patchouli genome was carried out by running RepeatModeler (http://www.repeatmasker.org/RepeatModeler/) and RepeatMasker (http://repeatmasker.org) (Supplementary Figure S2). The patchouli-specific *de novo* repeat libraries were constructed by combining results from LTR_STRUC^27^ and RepeatModeler with default parameters. The consensus sequences in patchouli-specific *de novo* repeats libraries and their classification information were further combined with library from RepeatMasker and then used to run RepeatMasker on the assembled scaffolds, followed by further tandem repeats identification using TRF^28^. *Sa. miltiorrhiza, Se. indicum, Mi. guttatus,* and *U. gibba* genomes were annotated with the same pipeline (Supplementary Table S7–S8).

### Gene annotation

Transcriptome alignment, *de novo* gene prediction, and sequence homology-based predictions were used for gene prediction (Fig. 1).

Before transcriptome alignment, RNA-Seq reads were assembled into transcripts. To capture more protein-coding genes, we also included three samples from our previous paper^29^. Illumina raw reads (Supplementary Table S2) were filtered with following steps before transcriptome *de novo* assembly. Read pairs with adapter contamination, read pairs with N contents larger than 3% and read pairs with low quality bases (quality<20) larger than 20% were further removed. Finally, reads with potential low-quality regions were trimmed by applying Trimmomatic (v3.20)^21^. Reads with a quality score below 15 at the beginning or at the end were also trimmed off and reads containing 3’ or 5’ ends with an average quality score dropping below Q20 in a 4-base pair sliding window were trimmed. Any reads becoming shorter than 32 bp were excluded for further assembly (Supplementary Table S9). After trimming, all the clean reads were used for assembly using Trinity (version2.0.3)^30^ under default parameters(Supplementary Table S10). Then assembled transcripts were aligned to the genomes to obtain gene structure annotation information using PASA^31^.

For *de novo* gene prediction, SNAP^32^, GeneMark-ET^33^ and Augustus^34^ were used to predict genes on transposable-elements-hard-masked genome sequences, and the high quality data set for training these *ab initio* gene predictors was generated by PASA^31^. For sequence homology based gene prediction, proteins sequences from SwissProt plants database and five organisms (*A. thalian*a TAIR10, *V. vinifera* IGGP_12×, *Se. indicum* BGIv1.0, *So. tuberosum* PGSC_DM_v4.03 and *U. gibba* COGEv4.1) were incorporated into MAKER2^35^ to generate homology gene structures. All predicted gene structures were integrated into that consensus gene models using MAKER2^35^.

To evaluate whether our gene models were contaminated by large number of transposable element related proteins, the proteomes of patchouli and the well annotated model organism rice (Ensembl 34) were BLASTed against the sequences of the transposable elements (protein and nucleotide sequences) in RepBase^36^ (Supplementary Table S11). To evaluate whether the predicted protein-coding genes were fragmental or complete, a simple method is to compare their full length homologues. Therefore, we blasted the proteome of patchouli against the SwissProt database^37^ and the top hits of each protein were extracted. The length ratio was computed as the length of the patchouli protein divided by the length of its corresponding SwissProt homologous, and their frequency were plotted (Fig. 2b and Supplementary Table S12). The distribution of each elements of protein-coding genes were also plotted (Fig. 2c).

To determine the function of the gene models, a BLASTP^38^ search (with stringent criteria: e-value ≤ 1e^-5^, identity >  = 30% and coverage >  = 50%) was performed against protein databases, including NR (non-redundant protein sequences in NCBI), SwissProt^37^, and KEGG^39^ (Supplementary Table S13). The resulting NR BLASTP hits were processed by BLAST2GO^40^ to retrieve associated Gene Ontology (GO) terms^41^ describing biological processes (BP), molecular functions (MF), and cellular components (CC). The motifs and domains of each gene model was predicted by InterProScan^42^ (version 4.8) against public protein databases, including ProDom^43^, PRINTS^44^, Pfam^45^, SMART^46^, PANTHER^47^, PROSITE^48^ and TIGR^49^.

### Identification of gene families

Protein sequences of ten plant species were downloaded from JGI (release version 12) or their official web (Supplementary Table S14). Only the longest transcript was selected for each gene locus with alternative splicing variants. The genes with less than 50 amino acids were removed. Self-to-self alignments was conducted for pooled protein sequences using BLASTP^38^ with an E-value of 1e^-5^, and low quality hits (identity < 30% and coverage < 30%) were removed. Orthologous groups were constructed by ORTHOMCL^50^ v2.0.9 using default settings based on the filtered BLASTP results (Supplementary Table S15). The genes that could not be clustered into any gene family and that only one species exists are species-specific. Statistically significantly over-represented GO terms^41^ among these patchouli specific genes were identified (Supplementary Table S16) using BiNGO^51^ in Cytoscape^52^ with hypergeometric test. The whole GO annotations of patchouli genes was set as reference, and Benjamini & Hochberg correction was applied.

### Phylogenetic tree construction and divergence time estimation

Each proteome was BLASTed against to *V. vinifera* with an E-value ≤ 1e^-5^. Reciprocal best hits (RBHs) in each pair were obtained and the gene families with all the eleven-species present were kept. The protein sequences from each family were aligned using MUSCLE v3.8.31^53^ with default parameters, and the corresponding CDS alignments were back-translated from the corresponding protein alignments. The conserved CDS alignments were extracted by Gblocks^54^, and the remained CDS alignments of each family were used for further phylogenomic analyses. For phylogenetic tree construction, CDS alignments of each single family were concatenated to generate a matrix of 3,511,077 unambiguously aligned nucleotide positions. 4DTV sites were extracted from these super-genes, and Mrbayes3.22^55^ was used to generate Bayesian tree with GTR + I + Γ model using 4DTV sites. The MCMC process was run 1,000,000 generations, and trees were sampled every 100 generations with first 2,500 samples drop. The concatenated supergenes were separated into three partitions, corresponding to the 1^st^, 2^nd^ and 3^rd^ codon site in the CDS. Super-genes constructed from full-length, 1^st^ codon, 2^nd^ codon and 4DTV sites were also subject to RAxML^56^ to generate maximum likelihood tree with GTR + I + Γ model (Supplementary Figure S3).

Considering that the evolutionary rates are vastly different at the different codon positions, the three codon positions of the concatenated supergene were treated as three different partitions. Divergence times were estimated under a relaxed clock model using the MCMCTREE program in the PAML4.7 package^57^. “Independent rates model (clock = 2)” and “JC69” model in MCMCTREE program were used. The MCMC process was run for 6,000,000 iterations, after a burn-in of 2,000,000 iterations. We ran the program twice for each data type to confirm that the results were similar between runs. The chronogram was produced using FigTree v1.4.0 (http://tree.bio.ed.ac.uk/) with the first run. We selected 5.09-10.25 Myr and 110-124 Myr as the lower and upper boundaries for the tomato-potato and tomato-Arabidopsis respectively^58^.

### Polyploidization analyses

We used MCScanX^59^ to detect syntenic blocks (regions with at least five collinear genes) and duplication levels (duplication depth). Synonymous substitutions per synonymous site (Ks) for syntenic genes were calculated using YN00 from PAML package^57^. Paralogs and orthologs tracing to pan-eudicot γ triplication were fetched from MCScanX collinearity results. We identified OrthoMCL gene families consisting of all the 11 species. For each such family, we kept all the paralogs from patchouli, while only kept the longest one paralogs for other species. The protein sequences from each such family were aligned using MUSCLE v3.8.31^53^ with default parameters, and the corresponding CDS alignments were obtained from the corresponding protein alignments using the PAL2NAL^60^. The maximum likelihood tree for these families were generated by the RAxML^56^ to with GTR + I + Γ model, and were filtered if they conflict with species tree. We then used MCMCTREE program in the PAML4.7 package^57^ to estimate the divergence times of the genes in these families. MCMCTREE was run as described above except that the CDS alignments were not partitioned. Finally, the divergence time of patchouli—*Sa. miltiorrhiza* and the divergence time of patchouli paralogs oldest clade were extracted plotted.

### Analyses genes related to biosynthesis of patchouli oil

Protein sequences of five patchouli *TPS* genes were downloaded from NCBI (AY508726.1, AY508728.1, AY508729.1, AY508730.1, and AY508727.1). Longest ORF in each gene loci in the patchouli gene set was selected as representative sequence, and then representative sequences were BLASTed against to the five reference TPS proteins with e-value of 1e-2. Blast hits were further annotated by PFAM database using IPRSCAN5. If the candidate presents both the two TPS-related domains (PF03936: terpene synthase family, metal binding domain; PF01397: terpene synthase, N-terminal domain), it is classified as full length, while if the candidate presents only one of them, it is classified as partial. Similar methods were applied to the identification of *TPS* genes in the other eight species, including *A. thaliana, Mi. guttatus, Sa. miltiorrhiza, Se. indicum, So. lycopersicum, So. tuberosum, U. gibba,* and *V. vinifera* (Supplementary Table S17). The protein sequences of full length *TPS* genes identified above were aligned using MUSCLE v3.8.31^53^ with default parameters, and the corresponding CDS alignments were back-translated from the corresponding protein alignments using PAL2NAL^60^. RAxML^56^ was used to generate maximum likelihood with GTR + I + Γ model and 100 bootstraps. Trees were plotted by the iTOL (https://itol.embl.de/). The protein-coding gene annotations were updated with UTRs and models for alternative splicing using PASA pipeline (https://pasapipeline.github.io/). Then, genes and transcripts were quantified using align_and_estimate_abundance.pl provided by the Trinity^61^ package (version 2.2.0). The Pearson correlation of samples were calculated (Supplementary Figure S4).

### Code Availability

Custom codes used for dataset analysis were stated in the methods section. Software and their used versions were described in methods.

## Data Records

All of the raw reads for the patchouli genome have been deposited in the NCBI Sequence Read Archive (SRA) (Data Citation 1). All of the raw reads for the patchouli transcriptome have been deposited in the NCBI SRA (Data Citation 2). The genome assemblies have been deposited at GenBank (Data Citation 3). Other data records presented in this descriptor are available online from Figshare (Data Citation 4). The genome assemblies deposited in GenBank are also presented in Figshare (File 1, genome assemblies, Data Citation 4). The repetitive elements are recorded in GFF3 format (File 2, repeat annotations, Data Citation 4). The protein-coding gene annotation results (File 3, predicted coding genes, Data Citation 3) contain the coordinates of genes (GFF3 format) coding sequences (CDS) and protein sequences (FASTA format). The ortholog groups file is presented as original outputs by the OrthoMCL (File 4, OrthoMCL gene families, Data Citation 4). The intra- and inter-species collinear blocks (File 5, MCScanX results, Data Citation 4) are in text format and html format generated by MCScanX. The Figshare also includes the updated gene models with alternative splicing transcripts using RNASeq data as well as their expression levels in TSV format (File 6, RNASeq results, Data Citation 4). The full-length and partial TPS genes with their Accessions and classification are presented in XLS/TSV format (File 7, TPS genes, Data Citation 4).

## Technical Validation

Using a whole-genome shotgun strategy and the Illumina HiSeq platform, we generated ~681 gigabases (Gb) of genomic short sequences with ~355X coverage. The assembled genome is ~1.91 GB with a scaffold N50 value of 699,555 bp (Table 1). Approximately 90% of the genome sequence was contained in the 3,543 longest scaffolds ( > 74 kb), with the largest spanning 9 Mb. The distribution of GC contents and sequencing depths revealed a quite normal GC contents and sequencing depth (Fig. 2a). The genome size of patchouli was estimated ranging from 1.78 GB to 2.38 GB (Supplementary Table S4). Both the assembled and estimated genome size is the largest among the closest relatives in *Lamiales* with genome available (Table 1), reflecting potential repeats expansion and/or polyploidization, which have been identified in in many plants^2–5^. To assess assembly quality, we used a core eukaryotic gene mapping approach (CEGMA)^62^ to identify the core genes in the patchouli genome assembly; and 237 core eukaryotic genes of 248 (95.56%) were found in the assembly (Supplementary Table S6). We also evaluated the genome using sets of benchmarking universal single-copy orthologs (BUSCO) with genome mode^63^. For the total 1440 BUSCO groups searched, 136 (9.4%) were missing, revealing a completeness score of around 90.6%. These CEGMA and BUSCO results indicated that most of the evolutionarily conserved core gene set was present in the assembly suggesting a high quality assembly. Interestingly, among the 1288 (89.5%) complete BUSCO groups presented in patchouli genome, 969 (67.3%) were duplicated, suggesting the duplication of conserved core genes, which may result from whole-genome duplication.

We predicted 110,850 protein-coding gene models using a combination of *ab initio* prediction, homology alignment and transcript evidence assembled from RNA-Seq from multiple tissues using maker2 (Fig. 1). As the number of protein-coding genes is much larger than its close relatives (Table 1), series analyses were conducted to validate the annotation quality. To evaluate whether our gene models were contaminated by large number of TEs related proteins, the proteomes of patchouli and the well annotated model organism *Oryza indica* (Ensembl 34) were aligned to TEs from RepBase^36^, and a similar percentage of potential TE-related genes in these two species was observed. To determine whether these gene models were functional, we aligned the patchouli proteome to the functional databases and annotated the domains using InterProScan^42^. Using stringent filtering criteria, a total of 71.17%, 42.67% and 28.92% of the gene models were annotated using the NR, SwissProt^37^, and KEGG^39^ respectively. In addition, 87.66% of the gene models were annotated with to domains. In total, the combined annotation procedure was able to assign annotations for 89.55% of the gene models. To evaluate whether the predicted protein-coding genes were fragmental, which result in more predicted gene number by breaking complete gene into more fragments, the ratios of patchouli protein lengths to their SwissProt homolog’s lengths were plotted and results shown that patchouli has similar distribution pattern when compared to its close relatives (Fig. 2b). In addition, patchouli also has similar distribution patterns of the CDS length and the CDS numbers of each gene to the distribution patterns of those elements from more close relative species (Fig. 2c). Together, these results revealed the high annotation accuracy, and the number of gene models, which is two times (*O. europaea*^2^) to 4 times (*Se. indicum*^4^) more than its close relatives (Table 1), is strongly suggestive of polyploidization.

A total of 770 Mb repetitive elements were annotated, predominantly contributed by transposable elements (TEs) and accounting for 43.68% of the assembly genome. When compared with another four *Lamiales* species genome sequence, although the percentage of repetitive elements is higher than those of *Sa. miltiorrhiza*, but it is similar and even lower to those of *Se. indicum* and *Mi. guttatus*, which all have much smaller genome size (Table 1). Moreover, the genic regions, the repetitive sequences and the other genomic sequences (non-genic and non-repetitive sequences) are all expanded proportionally when compared with other *Lamiales* relatives (Fig. 2d). Altogether, these results indicate that the genome size expansion in patchouli mainly resulted from the presence of polyploidization rather than the expansion of repetitive sequences.

Predicted protein sequences of patchouli and the complete proteome of another ten plant species were clustered into 33,517 gene families by OrthoMCL v2.0.9^50^ following self–self–comparisons with the BLASTP program. Although much larger genome size and gene set compared to other species, patchouli has much lower proportion of un-clustered genes (Fig. 2e), reflecting the high quality of annotation. Notably, much larger average number of genes per gene family of patchouli is also strongly suggestive of polyploidization, when compared to other species, even much larger than that of *U. gibba*, which was demonstrated to undergo three rounds of WGD since common ancestry with *So. lycopersicum* and *V. vinifera*^5^.

**Figure 3:**
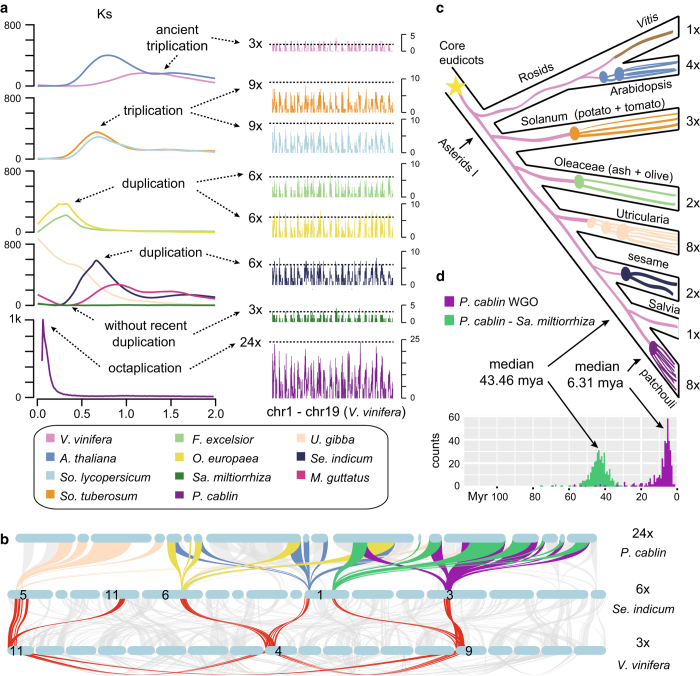
Polyploidization events in patchouli and its relatives. (**a**) Distribution of Ks for paralogs identified in co-linear regions of each selected species (left) and their inferred duplication level (right). (**b**) Co-linear alignment blocks of patchouli-*Se. indicum* and *Se. indicum*-*V. vinifera* (grey lines). Highlighted regions (colour lines) trace to a common ancestor before the pan-eudicot hexaploidy. (**c**) Palaeohistory highlighting the phylogenetic position of patchouli. (**d**) Estimated time of patchouli polyploidization event. WGO: whole-genome octuplication.

Distributions of synonymous substitutions per synonymous site (Ks) (Fig. 3a) for paralogous patchouli genes showed a clear and sharp peak at Ks ≈ 0.01. More interestingly, there’s no any observable peak in *Sa. miltiorrhiza*, which is the close relative of patchouli^64^. Moreover, the comparison of distributions of Ks for *Se. indicum* and patchouli also indicated that there’s no shared polyploidization event except the ancestral WGT shared by all core eudicots (WGT-γ). These results indicated a recent and exclusive polyploidization event. The hexaploidy *V. vinifera* (Fig. 3a) is considered to be the closest modern representative of the ancestral eudicot karyotype consisting of 7 (pre-γ ancestor) or 21 (post-γ ancestor) protochromosomes^65^, therefore, is used as reference genome to assess the palaeohistory of eudicots (Fig. 3). Individual *V. vinifera* chromosome segments generally have duplication level of three (number of syntenic segments in each co-linear region) (Fig. 3a). The duplication levels of both *So. tuberosum*^66^ and *So. lycopersicum*^67^ are nine, which is consistent with the well demonstrated *Solanum* WGT after the pan-eudicot γ triplication. Although polyploidization event is expected according to previous clues, the duplication level in patchouli reaches as large as 24, indicating that since pan-eudicots γ triplication, totally octuplication occurred in patchouli. Using *Se. indicum*^4^ as bridge, which is phylogenetic close relatives to patchouli and chromosome level assembly, we confirmed the WGO event with orthologous and paralogous genes tracing (Fig. 3b). Considering that the Ks distribution of patchouli is sharp rather than flat as that of *U. gibba*, which has undergone three sequential WGD events, the putative octuplication in patchouli may be either multiple closely spaced independent WGD events or more parsimoniously a single WGO event.

Compared with independently polyploidy *U. gibba*, which shows extremely fractionated gene loss^5^, the patchouli genome shows much less gene loss. Assuming a similar initial protein-coding gene repository of last common ancestor of patchouli and *U .gibba*, the newly-formed polyploidies would also have similar gene number when considering the same polyploidization level^5^ (Fig. 3c). However, the remaining protein-coding gene repository in patchouli is 3~4 times more than those in *U. gibba*, with the count of 110,850 in patchouli and 30,689 in *U. gibba*^5^ (Table 1). Bayesian molecular dating was adopted to estimate the WGO event in patchouli, and the paralogs in the duplicated gene family dated the polyploidization event ~6.31 MYA (Fig. 3d). The lower fraction of gene loss may result from relatively short age of this WGO event, therefore offering a unique opportunity to study the retention of whole-genome duplication.

**Figure 4:**
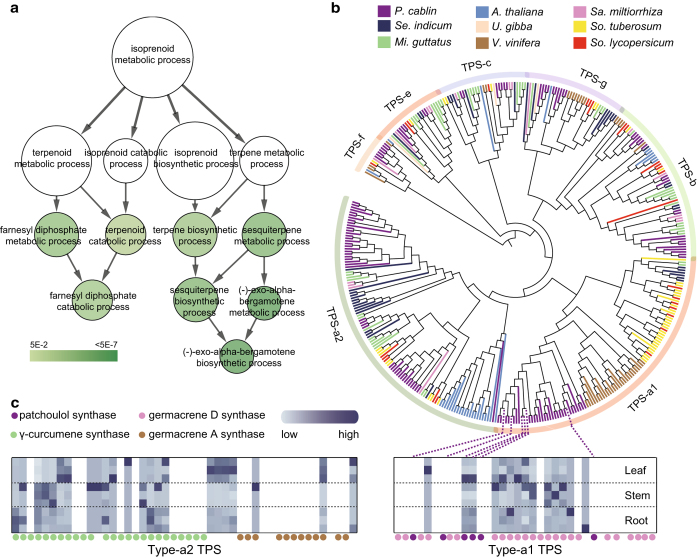
Biosynthesis of patchouli oil in the patchouli. (**a**) Over-represented gene ontology of patchouli-specific gene families. Each circle represents one term and its size is proportion to gene number. (**b**) Phylogeny of putative full-length TPSs from the nine sequenced plant genomes. Based on the phylogeny and functions of known TPSs, six subfamilies of TPSs are recognized, and the TPS-a subfamily is further divided into two groups, a1 and a2. (**c**) Expression of type a1 and a2 TPS genes in the patchouli. Purple dotted lines bridge the phylogenetic position and their expression of the six-full length patchoulol synthases (PTSs). Blank columns indicate that these genes were not transcribed in any of three tissues.

Patchouli oil is complex like many essential oils, but distinct because it consists largely of sesquiterpenes hydrocarbons, which including α-/β-/γ-patchoulenes, α-bulnesene, α-guaiene and seychellene, with structures clearly related to patchoulol and sesquiterpenes with unrelated structures like pogostone, trans-β-caryophyllene, α-humulene and γ-curcumene^68^. To explore the characteristics of patchouli oil biosynthesis, we first identified 4,602 patchouli-specific gene families consisting of 18,781 genes. Species-specific genes can confer unique molecular foundation for species-specific phenotypes. Indeed, these genes are over-represented in isoprenoid metabolic process (Fig. 4a) and acyl-CoA metabolic process that provides raw materials for isoprenoid synthesis (Supplementary Table S16), which suggests the novelty of patchouli oil metabolism in patchouli.

Then, we identified 268 terpene synthases (TPSs) gene loci in the patchouli genome (Supplementary Table S17), as TPSs form an important gene family that responses for the synthesis of the various terpene molecules^69^. Of these TPSs gene loci, 131 loci are putatively full length with the presence of both the two *TPS*-related domains. All the six currently recognized angiosperm *TPS* subfamilies are present in patchouli (Fig. 4b). Classification based on known plant homologues reveals that the subclass TPS-a (putative sesquiterpenes synthases^69^) represents only 23.5% of the *Sa. miltiorrhiza* TPS family whereas this subclass represents 61.8% of the patchouli TPS family. More interestingly, most of the type-a *TPS* members were transcribed in at least one of the three tissues, and majority of this subclass consist of only four sesquiterpenes synthases (Fig. 4c). Among these sesquiterpenes synthases, the patchoulol synthase (*PTS*) has been isolated and characterized to be a multifunctional enzyme that synthesizes 14 kinds of sesquiterpenes, including patchoulol^68^, however, only two of them were detected in our transcriptome (Fig. 4c). These results suggest highly specialization of sesquiterpenes synthases that produce C15 terpenoids present in patchouli oil.

In summary, this paper released two important types of genomic resources for the patchouli, genome sequencing data and transcriptome sequencing data. This is also the first release of octaploid medicinal genome sequences with high assembly and annotation quality.

## Additional information

**How to cite this article**: He, Y. *et al*. Building an octaploid genome and transcriptome of the medicinal plant *Pogostemon cablin* from Lamiales. *Sci. Data*. 5:180274 doi: 10.1038/sdata.2018.274 (2018).

**Publisher’s note**: Springer Nature remains neutral with regard to jurisdictional claims in published maps and institutional affiliations.

## Supplementary Material



Supplementary Information

## Figures and Tables

**Table 1:** Statistics of plant genomes investigated in study.

Species	Genome Size(Mb)	Ploidy*	Scaffold N50 (Kb)	Contig N50 (Kb)	#Gene	Repeats	Reference
*P. cablin*	1,916	x8	699Kb	34.7Kb	110,850	43.68%	this
*Sa. miltiorrhiza*	641	x1	1.2 Mb	82.8 kb	34,598	29.38%	^64^
*Mi. guttatus*	322	x4	1.12 Mb	45.5 kb	31,820	55.77%	^10^
*Se. indicum*	274	x2	2.1 Mb	52.2 kb	27,148	44.59%	^4^
*U. gibba*	102	x8	3424Kb	33.1 kb	30,689	0.67%	^5^
*O. europaea*	1,311	x4	443Kb	52.4 kb	56,349	nd^∗∗^	^2^
*F. excelsior*	867	x4	104Kb	24.9 kb	38,852	nd	^3^
*So. lycopersicum*	760	x3	chromosome-level		34,727	nd	^67^
*So. tuberosum*	727	x3	chromosome-level		39,031	nd	^66^
*V. vinifera*	487	x1	chromosome-level		30,434	nd	^65^
^∗^Post-γ: after the ancestral whole-genome triplication shared by all core eudicots. ^∗∗^nd: not determined in this paper.							
