# Supplementary material for: Building an octaploid genome and transcriptome of the medicinal plant Pogostemon cablin from Lamiales
Source: Sci Data. 2018 Dec 11;5:180274. doi: 10.1038/sdata.2018.274 (PMC6289116; doi:10.1038/sdata.2018.274)

Building an octaploid genome and transcriptome of the medicinal plant Pogostemon cablin from Lamiales

Supplementary information

Contents

Table S1 .... **2**
Table S2 .... **2**
Table S3 .... **3**
Table S4 .... **3**
Table S5 .... **3**
Table S6 .... **4**
Table S7 .... **4**
Table S8 .... **4**
Table S9 .... **5**
Table S10 .... **5**
Table S11 .... **5**
Table S12 .... **6**
Table S13 .... **6**
Table S14 .... **6**
Table S15 .... **6**
Table S16 .... **7**
Table S17 .... **9**
Figure S1 .... **9**
Figure S2 .... **9**
Figure S3 .... **10**
Figure S4 .... **11**

Table S1. Raw sequencing data generated for the *P. cablin* genome sequencing project. Insert sizes include paired-end read lengths. Coverage was calculated under the assumption of a genome size of 1.91 GB.

Insert size	library	Length	Read pairs	Total bases for each library	Depth (X)
250bp	250-1	150	217,164,765	65,149,429,500	34.11
	250-2	150	230,674,280	69,202,284,000	36.23
500bp	500-1	150	174,837,947	52,451,382,100	27.46
	500-2	150	189,701,658	56,910,497,400	29.80
700bp	700-1	150	183,866,822	55,527,780,244	29.07
	700-2	150	198,809,125	60,040,355,750	31.43
2k	2k-1	150	222,438,081	66,731,424,300	34.94
	2k-2	150	224,840,696	67,452,208,800	35.32
5k	5k-1	150	213,051,314	63,915,394,200	33.46
	5k-2	150	237,263,219	71,178,965,700	37.27
10k	10k	150	120,452,872	36,135,861,600	18.92
		150	56,296,770	16,889,031,000	8.84
	Total		2,269,397,549	681,584,614,594	355.56

Table S2. Statistics for RNA-Seq raw data

Sample	read pairs	bases	source
L1	32,796,719	9,904,609,138	this project
L2	27,105,784	8,185,946,768	this project
L3	31,460,420	9,501,046,840	this project
R1	28,107,730	8,488,534,460	this project
R2	29,039,044	8,769,791,288	this project
R3	29,356,410	8,865,635,820	this project
S1	34,101,559	10,298,670,818	this project
S2	32,099,007	9,693,900,114	this project
S3	29,552,169	8,924,755,038	this project
HY	69,311,944	13,862,388,800	PRJNA272750
HY-leaf	12,629,450	2,525,890,000	PRJNA272750
HY-stem	13,256,492	2,651,298,400	PRJNA272750

Table S3. Clean sequencing data used by the *P. cablin* genome sequencing project. Coverage was calculated under the assumption of a genome size of 1.91 GB.

Insert size	library	total reads	total bases	Depth (X)
250bp	250-1	196,146,187	38,406,542,677	20.11
	250-2	206,523,208	40,420,275,027	21.16
500bp	500-1	265,590,640	36,157,632,656	18.93
	500-2	281,714,402	38,143,061,348	19.97
700bp	700-1	305,369,016	40,085,143,976	20.99
	700-2	310,623,006	40,376,610,478	21.14
2k	2k-1	205,817,632	20,216,322,835	10.58
	2k-2	213,867,846	20,967,662,162	10.98
5k	5k-1	175,651,486	17,252,388,467	9.03
	5k-2	200,234,146	19,468,150,987	10.19
10k	10k	76,045,382	7,503,439,796	3.93
		46,356,790	4,648,950,486	2.43
Total		2,483,939,741	323,646,180,895	168.85

Table S4. Patchouli genome size estimated by different methods and datasets

Method	Kmer	Library	Total bases	Estimated genome size	depth(X)
SOAPec	17	250bp	100,107,225,904	2,348,706,702	42.62
SOAPec	17	500bp	76,466,100,293	2,383,224,406	32.09
SOAPec	17	700bp	82,951,726,672	2,287,372,764	36.27
KmerGenie	127	250bp+500bp	153,127,511,708	1,782,124,451	85.92
Jellyfish	27	250bp	100,107,225,904	1,957,022,783	51.15
Jellyfish	31	250bp	100,107,225,904	1,998,448,375	50.09
Jellyfish	27	250bp+500bp	153,127,511,708	1,844,824,309	83.00
Jellyfish	31	250bp+500bp	153,127,511,708	1,899,003,262	80.64

Table S5. Summarization for the de novo assemblies of patchouli. N50 is the minimum sequence length of 50% of the entire assembly.

	scaffold		contig	
	length(bp)	number	length(bp)	number
Max length	9,075,775		657,349	
N10	3,307,676	43	96,639	1,330
N30	1,389,726	230	54,762	6,370
N50	699,555	636	34,743	14,523
N70	354,136	1,412	20,071	27,840
N90	74,241	3,543	6,192	57,625
Total length	1,916,863,142		1,763,018,886	
number>=1000bp	41,734		130,148	
GC rate	0.329		0.35	

Table S6. Statistics of the completeness of the *P. cablin* genome using CEGMA. These results are based on the set of genes selected by Genis Parra. Prots = number of 248 ultra-conserved CEGs present in genome. %Completeness = percentage of 248 ultra-conserved CEGs present. Total = total number of CEGs present including putative orthologs. Average = average number of orthologs per CEG. %Ortho = percentage of detected CEGS that have more than 1 ortholog.

	#Prots	%Completeness	-	#Total	Average	%Ortho
Complete	226	91.13	-	905	4.00	96.90
Group 1	61	92.42	-	227	3.72	96.72
Group 2	49	87.50	-	199	4.06	97.96
Group 3	57	93.44	-	237	4.16	96.49
Group 4	59	90.77	-	242	4.10	96.61
Partial	237	95.56	-	1027	4.33	97.47
Group 1	63	95.45	-	272	4.32	98.41
Group 2	52	92.86	-	222	4.27	98.08
Group 3	61	100.00	-	268	4.39	96.72
Group 4	61	93.85	-	265	4.34	96.72

Table S7. Repeat contents of selected genomes. * Extreme genome size reductions have been reported in *Utricularia gibba*; therefore it's not used in the downstream analysis in this section.

Species	Genome Size	Count	Length (bp)	Repeat contents
Sa. miltiorrhiza	611,633,377	849,367	179,712,577	29.38%
Se. indicum	270,357,869	421,703	120,550,352	44.59%
P. cablin	1,763,018,886	2,346,338	770,110,585	43.68%
Mi. guttatus	289,885,078	505,356	161,665,292	55.77%
*U. gibba**	81,385,102	7,954	542,629	0.67%

Table S8. Repeat content (subtypes) of the selected genomes.

Species	Transposable Elements (TE)					Non-TE-Repeats
	DNA	LTR	LINE	SINE	Unknown	
Sa. miltiorrhiza	7.46%	13.04%	2.59%	0.26%	1.16%	4.87%
Se. indicum	15.83%	18.20%	5.17%	0.49%	2.10%	2.80%
P. cablin	8.91%	27.76%	1.86%	0.07%	1.35%	3.73%
Mi. guttatus	19.26%	28.13%	3.52%	0.48%	1.13%	3.25%

Table S9. Statistics for RNA-Seq clean data

Sample		read pairs	bases	Reference
L1	read 1	30,356,232	4,498,975,031	this project
L1	read 2	30,356,232	4,277,486,415	this project
L2	read 1	24,596,208	3,652,094,868	this project
L2	read 2	24,596,208	3,399,137,172	this project
L3	read 1	28,966,260	4,291,346,080	this project
L3	read 2	28,966,260	4,056,893,639	this project
R1	read 1	25,860,523	3,822,277,841	this project
R1	read 2	25,860,523	3,554,354,176	this project
R2	read 1	26,765,146	3,952,013,889	this project
R2	read 2	26,765,146	3,693,185,869	this project
R3	read 1	27,073,862	4,018,103,183	this project
R3	read 2	27,073,862	3,804,512,963	this project
S1	read 1	31,629,650	4,686,461,434	this project
S1	read 2	31,629,650	4,470,175,114	this project
S2	read 1	29,358,266	4,352,073,200	this project
S2	read 2	29,358,266	4,131,353,706	this project
S3	read 1	27,253,944	4,036,073,263	this project
S3	read 2	27,253,944	3,815,594,615	this project
HY	read 1	69,206,626	6,920,349,910	PRJNA272750
HY	read 2	69,206,626	6,907,109,489	PRJNA272750
HY-leaf	read 1	12,610,519	1,261,022,878	PRJNA272750
HY-leaf	read 2	12,610,519	1,258,902,448	PRJNA272750
HY-stem	read 1	13,237,378	1,323,705,569	PRJNA272750
HY-stem	read 2	13,237,378	1,321,457,809	PRJNA272750

Table S10. Statistics for transcriptome assemblies.

	Total trinity 'genes'	122,345
	Total trinity transcripts	207,932
	Percent GC	41.15
Stats based on ALL transcript contigs	Total bases	175,799,402
	Contig N10	3,194
	Contig N20	2,475
	Contig N30	2,006
	Contig N40	1,649
	Contig N50	1,345
Stats based on ONLY LONGEST ISOFORM per 'GENE'	Total bases	78,660,887
	Contig N10	3,319
	Contig N20	2,421
	Contig N30	1,833
	Contig N40	1,384
	Contig N50	976

Table S11. The number and percentage of genes in *P. cablin* that are aligned to RepBase transposable elements.

Species	# gene models	Blastp	Tblastn	Total	Percentage
Oryza indica	40,700	907	634	1541	3.79%
P. cablin	110,850	2081	1816	3897	3.51%

Table S12. The number and percentage of genes in *P. cablin* that are annotated by SwissProt.

Species	# gene models	Gene number	Percentage
Mi. guttatus	27,955	19,707	70.50%
P. cablin	111,160	72,441	65.17%
Se. indicum	27,148	17,037	62.76%
U. gibba	26,804	14,805	55.23%

Table S13. The numbers of proteins in *P. cablin* that are annotated.

# gene models	Total	SwissProt	KEGG	NR	GO	InterPro
110,850	99,270	47,304	32,068	78,888	63,062	97,170

Table S14. Information for species used for the gene family identification.

Latin name	Latin name (abbreviation)	Abbreviation	Data source
Pogostemon cablin	*P. cablin*	Pcab	this paper
Salvia miltiorrhiza	*Sa. miltiorrhiza*	Smil	ftp://202.203.187.112
Mimulus guttatus	*Mi. guttatus*	Mgut	http://phytozome.jgi.doe.gov/
Sesamum indicum	*Se. indicum*	Sind	http://www.ocri-genomics.org
Utricularia gibba	*U. gibba*	Ugib	http://genomevolution.org
Olea europaea	*O. europaea*	Oeur	http://denovo.cnag.cat
Fraxinus excelsior	*F. excelsior*	Fexc	http://ashgenome.org
Solanum lycopersicum	*So. lycopersicum*	Slyc	ftp://ftp.solgenomics.net
Solanum tuberosum	*So. tuberosum*	Stub	ftp://ftp.solgenomics.net
Arabidopsis thaliana	*A. thaliana*	Atha	ftp://ftp.arabidopsis.org
Vitis vinifera	*V. vinifera*	Vvin	http://www.genoscope.cns.fr

Table S15. Statistics of gene families identified by OrthoMCL. The '#genes' means that column is the number of genes in gene families. The '#family' means that column is the number of gene families. 'Unclustered' are the genes that could not clustered into any gene family. 'Unique' is the gene family that only one species exists. The 'Common' is the gene family all species present.

			clustered		Unique		Common		# genes
Species	#genes	#unclustered	#genes	#families	#families	#genes	#families	#genes	per family
Atha	26,769	3,695	23,074	12,565	820	3,009	5,373	10,008	1.836
Fexc	38,811	7,974	30,837	16,335	230	507	5,373	14,077	1.888
Mgut	27,948	3,671	24,277	14,413	452	1,431	5,373	10,187	1.684
Oeur	56,321	14,840	41,481	17,713	1,421	4,943	5,373	15,788	2.342
Pcab	110,835	15,547	95,288	19,113	4,602	18,781	5,373	32,365	4.986
Sind	27,127	3,426	23,701	13,583	338	2,059	5,373	9,936	1.745
Slyc	33,809	8,427	25,382	16,252	486	1,855	5,373	9,606	1.562
Smil	27,541	6,877	20,664	12,886	983	3,339	5,373	8,735	1.604
Stub	38,989	7,115	31,874	16,244	828	5,979	5,373	9,656	1.962
Ugib	29,287	7,466	21,821	10,457	536	6,509	5,373	8,596	2.087
Vvin	25,258	5,958	19,300	12,607	622	1,877	5,373	8,825	1.531

Table S16. Statistically significantly over-represented GO terms among the *P. cablin* specific genes. The significant level is set as 0.05.

	Groups	GO ID	Corrected p-value	Description
BP	-	15074	4.76E-09	DNA integration
BP	-	7060	1.36E-02	male meiosis chromosome segregation
BP	acyl-CoA metabolic process	6086	6.19E-03	acetyl-CoA biosynthetic process from pyruvate
BP	acyl-CoA metabolic process	6104	1.36E-02	succinyl-CoA metabolic process
BP	cellular amino acid metabolic process	6105	1.25E-03	succinate metabolic process
BP	cellular amino acid metabolic process	9082	3.51E-03	branched-chain amino acid biosynthetic process
BP	cellular amino acid metabolic process	9081	2.02E-02	branched-chain amino acid metabolic process
BP	chromatin	34728	3.44E-02	nucleosome organization
BP	chromatin	6334	3.44E-02	nucleosome assembly
BP	isoprenoid metabolic process	1901938	3.29E-05	(-)-exo-alpha-bergamotene metabolic process
BP	isoprenoid metabolic process	1901940	3.29E-05	(-)-exo-alpha-bergamotene biosynthetic process
BP	isoprenoid metabolic process	51762	5.52E-04	sesquiterpene biosynthetic process
BP	isoprenoid metabolic process	51761	5.52E-04	sesquiterpene metabolic process
BP	isoprenoid metabolic process	45338	1.72E-03	farnesyl diphosphate metabolic process
BP	isoprenoid metabolic process	46246	4.16E-03	terpene biosynthetic process
BP	isoprenoid metabolic process	45339	4.92E-03	farnesyl diphosphate catabolic process
BP	isoprenoid metabolic process	16115	2.69E-02	terpenoid catabolic process
BP	response to stimulus	2250	1.36E-02	adaptive immune response
BP	response to stimulus	2438	1.36E-02	acute inflammatory response to antigenic stimulus
BP	response to stimulus	2437	1.36E-02	inflammatory response to antigenic stimulus
BP	response to stimulus	2460	1.36E-02	adaptive immune response
BP	response to stimulus	2449	1.36E-02	lymphocyte mediated immunity
BP	response to stimulus	19724	1.36E-02	B cell mediated immunity
BP	response to stimulus	2526	1.36E-02	acute inflammatory response
BP	response to stimulus	2524	1.36E-02	hypersensitivity
BP	response to stimulus	16064	1.36E-02	immunoglobulin mediated immune response
BP	response to stimulus	16068	1.36E-02	type I hypersensitivity

CC	-	5742	1.44E-02	mitochondrial outer membrane translocase complex
CC	-	45254	1.44E-02	pyruvate dehydrogenase complex
CC	chromosomal part	44815	8.54E-03	DNA packaging complex
CC	chromosomal part	786	2.86E-02	nucleosome
CC	chromosomal part	1990104	2.86E-02	DNA bending complex
MF	-	19904	1.25E-03	protein domain specific binding
MF	-	16538	2.80E-02	cyclin-dependent protein serine/threonine kinase regulator activity
MF	ligase activity	16879	2.20E-02	ligase activity, forming carbon-nitrogen bonds
MF	ligase activity	16881	3.31E-02	acid-amino acid ligase activity
MF	lyase activity	10333	9.28E-13	terpene synthase activity
MF	lyase activity	16838	3.46E-10	carbon-oxygen lyase activity, acting on phosphates
MF	lyase activity	10334	6.73E-05	sesquiterpene synthase activity
MF	lyase activity	16835	4.92E-03	carbon-oxygen lyase activity
MF	lyase activity	16841	1.62E-02	ammonia-lyase activity
MF	lyase activity	4834	2.69E-02	tryptophan synthase activity
MF	O-methyltransferase activity	8171	1.75E-09	O-methyltransferase activity
MF	O-methyltransferase activity	4719	2.79E-03	protein-L-isoaspartate (D-aspartate) O-methyltransferase activity
MF	O-methyltransferase activity	47763	1.35E-02	caffeate O-methyltransferase activity
MF	O-methyltransferase activity	51998	1.44E-02	protein carboxyl O-methyltransferase activity
MF	O-methyltransferase activity	10340	1.44E-02	carboxyl-O-methyltransferase activity
MF	O-methyltransferase activity	30761	1.79E-02	8-hydroxyquercitin 8-O-methyltransferase activity
MF	oxidoreductase activity	4738	4.92E-03	pyruvate dehydrogenase activity
MF	oxidoreductase activity	4739	4.92E-03	pyruvate dehydrogenase (acetyl-transferring) activity
MF	oxidoreductase activity	4024	1.79E-02	alcohol dehydrogenase activity, zinc-dependent
MF	oxidoreductase activity	16649	2.86E-02	oxidoreductase activity, acting on the CH-NH group of donors
MF	oxidoreductase activity	4174	2.86E-02	electron-transferring-flavoprotein dehydrogenase activity
MF	peptidase activity	70001	4.92E-03	aspartic-type peptidase activity
MF	peptidase activity	4190	1.36E-02	aspartic-type endopeptidase activity
MF	RNA binding	70883	2.86E-02	pre-miRNA binding
MF	RNA binding	70878	2.86E-02	primary miRNA binding

Table S17. Gene copy numbers of TPS genes from plant species separated by subfamilies. F: full length; P: partial.

Species	total			TPS-a1		TPS-a2		TPS-b		TPS-c		TPS-e		TPS-f		TPS-g	
	F+P	F	P	F	P	F	P	F	P	F	P	F	P	F	P	F	P
A. thaliana	34	32	2	0	0	22	2	6	0	1	0	1	0	1	0	1	0
Mi. guttatus	57	51	6	2	0	14	4	12	0	10	2	9	0	0	0	4	0
P. cablin	268	131	137	35	30	46	40	20	59	7	3	6	0	7	1	10	4
Se. indicum	48	41	7	6	4	15	2	5	0	5	1	3	0	0	0	7	0
So. lycopersicum	45	26	19	7	7	5	1	7	7	1	0	2	3	1	0	3	1
Sa. miltiorrhiza	26	17	9	0	1	4	2	5	3	3	2	3	1	1	0	1	0
So. tuberosum	77	45	32	28	18	8	7	2	4	3	0	1	1	1	0	2	2
U. gibba	3	3	0	0	0	0	0	0	0	1	0	1	0	0	0	1	0
V. vinifera	95	47	48	28	27	0	0	5	9	2	0	1	0	3	1	8	11

Figure S1. Estimation of the *P. cablin* genome size using KmerGenie. (**a**) The estimated genome size reached the maximum value when the Kmer was 127. (**b**) The distribution of 127-mer. Colors of the fits: red is the fit of the complete statistical model of the histogram (erronous k-mers + genomic k-mers), while green are only the homozygous k-mers.

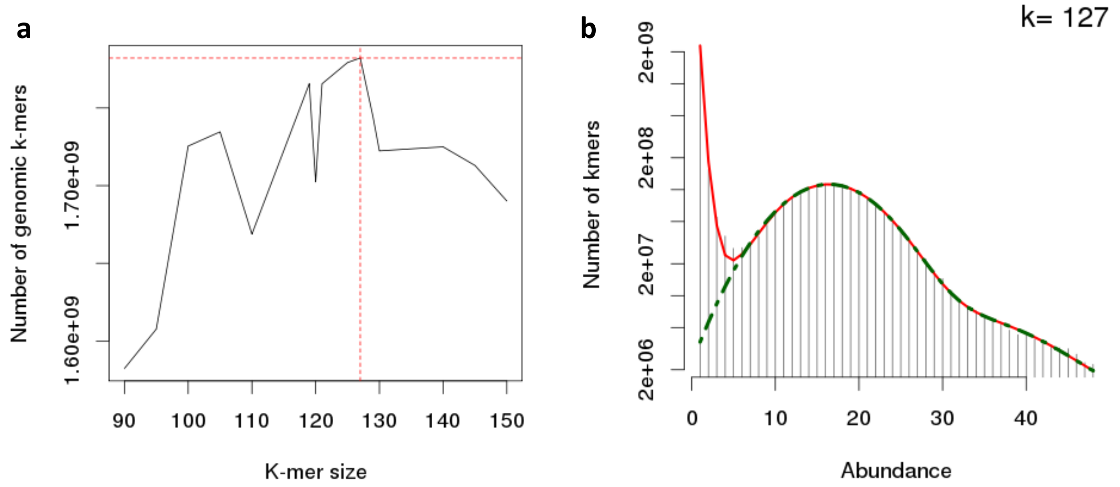

Figure S2. Pipeline of repeats annotation with *P. cablin*-specific repeat libraries.

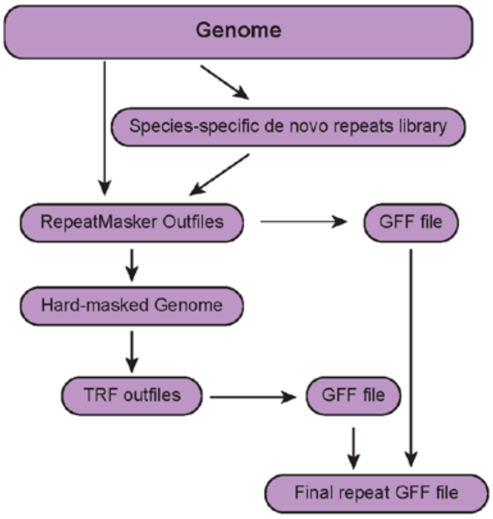

Figure S3.Maximum likelihood trees were reconstructed using RAXML using the first codon positions (**a**), the second codon positions (**b**), the 4DTv sites (**c**) and the full-length CDSs (**d**). Bayesian trees were reconstructed using MrBayes using the 4DTv sites (E). Each tree is rooted at the node where the asterids and rosids split. The abbreviations on each leaf are the same as those in Table S14. The bootstrap values (**a-d**) and posterior probabilities (**e**) are labeled on each node.

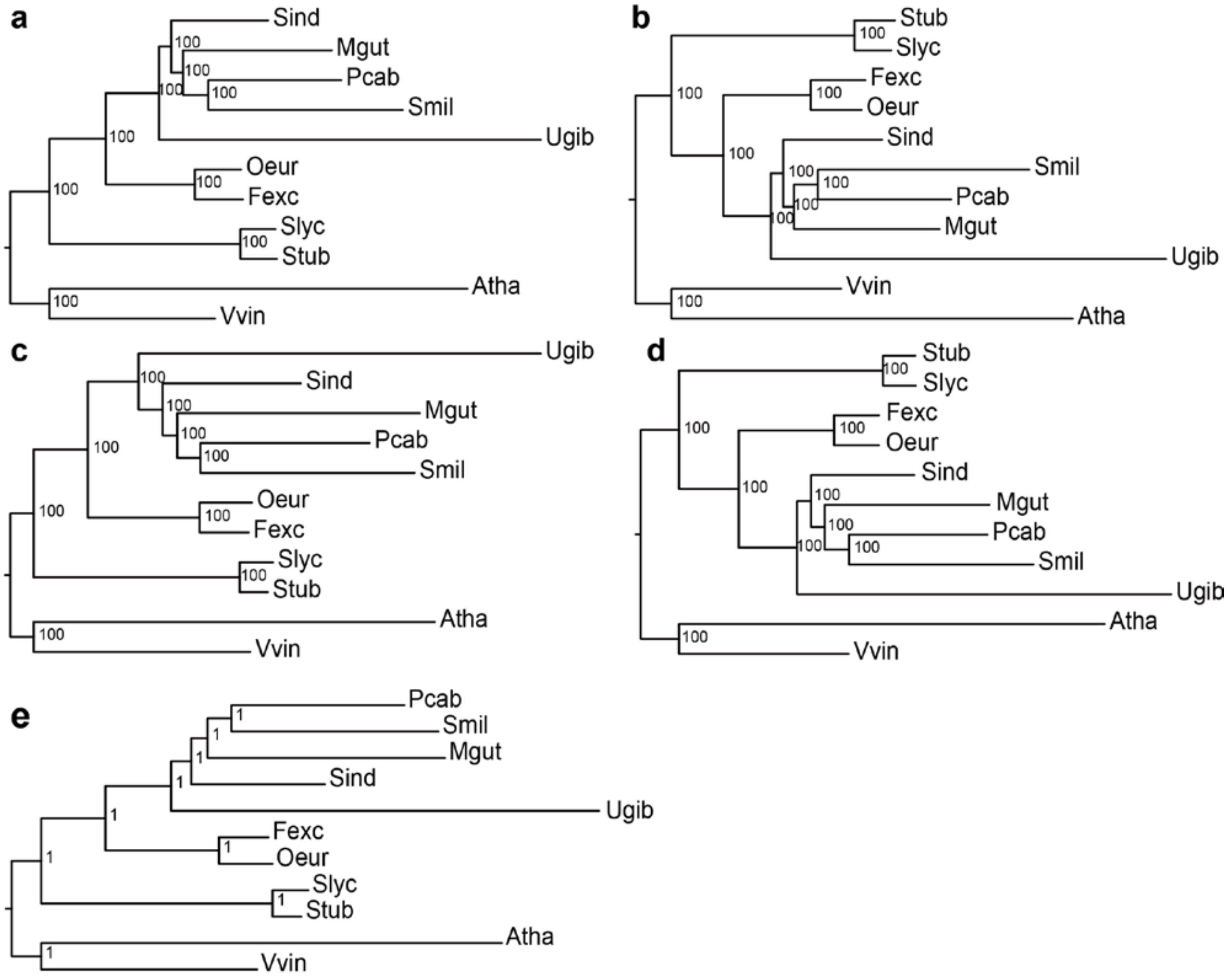

Figure S4.Correlation matrix of RNASeq samples.

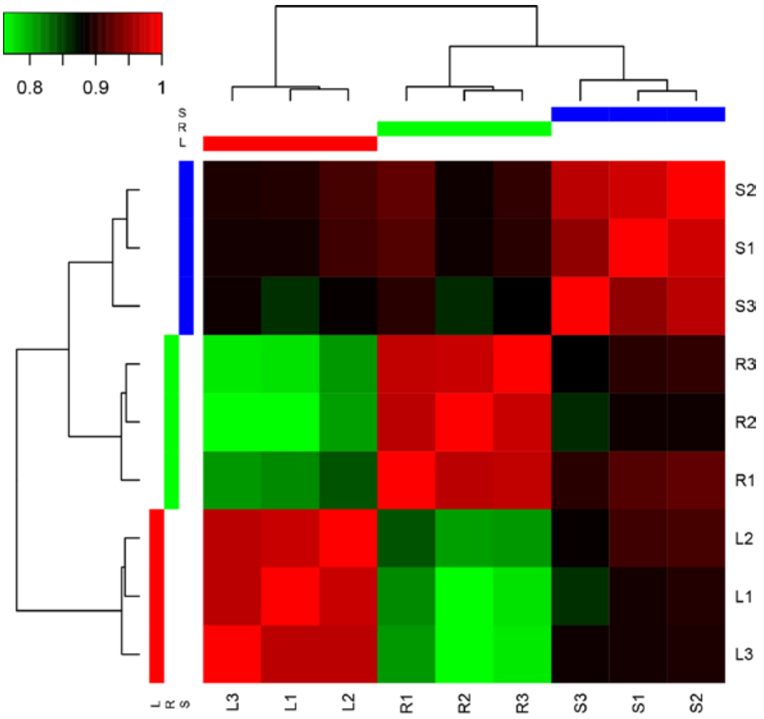

Supplement: Supplementary Information [file sdata2018274-s2.pdf]
